# Putative evolution of *Myxococcus fulvus* 124B02 plasmid pMF1 from a chromosomal segment in another *Myxococcus* species

**DOI:** 10.1128/spectrum.01206-24

**Published:** 2024-11-15

**Authors:** Shruti Jain, Gaurav Sharma

**Affiliations:** 1 Institute of Bioinformatics and Applied Biotechnology (IBAB), Bangalore, Karnataka, India; 2 Department of Biotechnology, Indian Institute of Technology Hyderabad, Sangareddy, Telangana, India; University of Mississippi, University, Mississippi, USA

**Keywords:** genomics, phylogeny, myxobacteria, synteny, evolution, plasmid

## Abstract

Myxobacteria or order Myxococcales (old nomenclature) or phylum Myxococcota (new terminology) are fascinating organisms well known for their diverse peculiar physiological, taxonomic, and genomic properties. Researchers have long sought to identify plasmids within these organisms, yet thus far, only two organisms from different families have been found to harbor a plasmid. This study delves into the putative evolution of one of these plasmids, i.e., pMF1 present in *Myxococcus fulvus* 124B02 in the suborder Cystobacterineae and family Myxococcaceae. Here, we first reannotated the pMF1 plasmid genome sequence and identified two additional open reading frames or putative genes which were not annotated until now. We further reported that all pMF1 plasmid genes depict homology with *Myxococcus stipitatus* CYD1 draft genome (contig 28) and a chromosomal segment of *M. stipitatus* DSM14675 in a syntenic manner, implying the presence of plasmid-like structure in *M. stipitatus* CYD1, integrated into its chromosome. To comprehend the relationship among these three species, we conducted phylogenetic analyses using 16S and concatenated housekeeping genes and genome-to-genome distance calculator (GGDC) analysis, which confirmed that *M. stipitatus* CYD1 is a distinct and novel species within the genus *Myxococcus*. Overall, this comparative genomic study sheds light on the putative emergence of the pMF1 plasmid from a common ancestor of closely related yet distinct species, *M. stipitatus* CYD1, possibly through the partition from its chromosome as a segment.

**IMPORTANCE:**

Myxobacteria are not well known to have plasmids. Until now, only two organisms have been shown to have plasmids, raising a pertinent question about how these plasmids evolved randomly within the phylum Myxococcota. The study presented in this manuscript delves into the emergence of the pMF1 plasmid found in *Myxococcus fulvus* 124B02, a member of the suborder Cystobacterineae and family Myxococcaceae. Our research addresses this intriguing topic of plasmid identification and evolution within myxobacteria, which are a group of fascinating organisms that have garnered significant interest due to their diverse physiological, taxonomic, and genomic properties.

## INTRODUCTION

Microorganisms are well known for being “ecosystem suppliers” as they are crucial for the environment and human life (1). Microbes have adapted themselves hugely according to their respective niches via diverse processes such as gene duplication, genome rearrangement, horizontal gene transfer, and gene loss (2). Myxobacteria, order Myxococcales (or phylum Myxococcota as of recent ICSP nomenclature) members, are gram-negative bacteria well known for their extraordinary multicellular social lifestyle (3). Their peculiar characteristics are influenced by their multicellular behavior, such as predation, cooperative movement, or social gliding motility (4) and multicellular structures known as fruiting bodies (5). 16S rRNA studies have shown that the myxobacteria group belongs to the class Deltaproteobacteria (6), and most of these organisms are aerobic except for facultative anaerobe *Anaeromyxobacter* spp. and strict anaerobe *Pajaroellobacter abortibovis*. Together with actinomycetes, *Bacillus* spp., and several fungi, myxobacteria are among the vast producers of diverse natural products such as polyketides, non-ribosomal polypeptides, terpenoids, phenylpropanoids, and alkaloids. Many of these compounds are effective against bacteria, viruses, fungi, cancer cells, and other microorganisms. Genetic engineering can play a pivotal role (7) in exploiting the potential of myxobacterial secondary metabolites by utilizing their plasmids (8). Until 2000, various myxobacteria species were screened, but no indigenous plasmid was found in any species. To re-evaluate whether myxobacterial organisms contain plasmids or not, about 150 strains belonging to the genus *Myxococcus* and a few genera *Corallococcus* (phylogenetically closely related genus of family Myxococcaceae and suborder Cystobacterineae) were rescreened, and a circular plasmid pMF1 was discovered in *M. fulvus* strain 124B02 (9). In 2021, another plasmid, pSa001, was identified from *Sandaracinus *sp. MSr10575, which belongs to the family Sandaracinaceae and suborder Sorangiineae (10). However, pMF1 remains the only plasmid known for the suborder Cystobacterineae and genus *Myxococcus*, the most studied model organism in the order Myxococcales.

*M. fulvus* strain 124B02 is an aerobic, gram-negative bacteria having slender rod-shaped vegetative cells with tapering ends and typical *Myxococcus*-type fruiting bodies containing spherical myxospores. *M. fulvus* 124B02 (*Mf*124B02) genome consists of a circular chromosome with a total length of 11.04 Mbp, 69.96% GC content, and 8,515 predicted coding sequences (11). Three completely identical 16S rRNA sequences are present in its genome in three different 16S-23S-5S operons located at 0.24, 7.35, and 10.84 Mb sites.

Plasmid pMF1 is a low copy number plasmid with a genome size of 18,634 bp. Its GC content (68.7%) is very similar to the Mf124B02 chromosome sequence (9). Twenty-three open reading frames (ORFs) have been predicted for this plasmid, of which 21 are on the sense strand and 2 (pMF1.19c and pMF1.20c) on the complementary strand. About 86% of the plasmid sequence consists of protein-coding sequences, although the function of 14 ORFs is mainly unknown (11). The region pMF1.13–pMF1.16 is the replication origin locus, out of which pMF1.14 is essential for plasmid propagation (9). pMF1.19–pMF1.20 region functions like a post-segregational killing system to improve plasmid maintenance (12). ORFs pMF1.19–pMF1.20 were previously described as SitAI toxin-immunity pairs, where the SitA toxin is delivered by the TraAB outer membrane exchange system (13). pMF1.21–pMF1.23 region helps retain plasmids' stability (14, 15). Until now, no apparent beneficial genes, such as genes coding for antibiotic resistance, virulence, or growth factors, have been identified, which are required for their persistence in the host (11). Research thus far has provided insights regarding replication locus, partitioning system, and post-segregational killing system in this plasmid. However, as the functions of most of the plasmid genes are not known, this study investigated in-depth annotations of this plasmid to find new putative genes and figured out how this plasmid sequence came into this organism (i.e., *Mf*124B02) or broadly within myxobacteria evolutionarily.

## MATERIALS AND METHODS

### Data collection

The complete genome (GCA_000988565.1) and plasmid pMF1 (EU137666.1) information for *Mf*124B02 were retrieved from the National Center for Biotechnology Information (NCBI) GenBank database (16). The plasmid pMF1 genome was reannotated using RAST (Rapid Annotations with Subsystem Technology) (17) and Prokka (version 1.14.6) (18) to certify that no other genes are present in the plasmid. The location of genes annotated from RAST, Prokka, and NCBI was compared to determine the unique number of genes in the plasmid (Table S1). The final translated coding sequence (CDS) file for the plasmid containing all the annotated genes was used for further exploration. A genome statistics table of *Mf*124B02 complete genome, plasmid (NCBI annotated), and merged plasmid annotation based on genome size, total number of genes, coding density, CDS (positive strand), CDS (negative strand), GC content, etc. was prepared (Table 1).

**TABLE 1:** Genome statistics of *M. fulvus* 124B02 chromosome, plasmid pMF1, and reannotated plasmid pMF1

Attribute	*M. fulvus* 124B02 chromosome	Plasmid pMF1	Reannotated plasmid pMF1
NCBI ID	CP006003.1	EU137666.1	EU137666.1
Genome size	10.7845 Mb	18.634 kb	18.634 kb
Coding density (%)	90.46	85.6	92.6
GC content (%)	70	68.7	68.7
CDS	8661	23	25
CDS (positive strand)	4163	21	23
CDS (negative strand or complement)	4498	2	2
Hypothetical protein	3226	16	18
Gene	8661	23	25
Pseudogene	0	0	0
Total RNA	92	0	0
rRNA	9	0	0
tRNA	79	0	0
ncRNA	3	0	0
tmRNA	1	0	0

### Function prediction of all annotated genes within plasmid pMF1

The input plasmid genome sequence was subjected to PLSDB, an open-source plasmid database (19), to find its homology with available plasmid sequences. To remove the possibility of plasmid’s sporadic or continuous homology within the *Mf*124B02 genome, plasmid pMf1 gene/protein sequences were subjected to standalone blastn/blastp (version 2.11.0) against *Mf*124B02 gene/protein sequences. To decipher the relationship of the plasmid with other organisms, blastp analysis (20) for all plasmid-translated CDS sequences was performed against the NCBI non-redundant (NR) database. Taxonomy information was obtained for each blast hit using the “efetch” tool and parsed with the result. Homologs and their corresponding taxonomy information of all hits were analyzed to determine if there is any synteny between the pMF1 plasmid and other organisms. *Myxococcus stipitatus* CYD1 (*Ms*CYD1) and *M. stipitatus* DSM 14675 (*Ms*DSM14675) translated CDS files were downloaded from the NCBI RefSeq database, and the individual database for both files was generated using makeblastdb. Standalone blastp (version 2.11.0) was performed to compare the plasmid pMF1 protein sequences against the *Ms*CYD1 and *Ms*DSM14675.

### Genome annotation of *Ms*CYD1 and *Ms*DSM14675

Contig 28 of *Ms*CYD1 was reannotated using RAST and Prokka (version 1.14.6), and the locations of all annotated genes using RAST, Prokka, and NCBI were compared followed by the identification of new open reading frames. All pMF1 translated proteins were subjected to blastp against *Ms*CYD1 proteins with 1e−10 e-value cutoff. The remaining plasmid and *Ms*CYD1 genes that did not show the homology were compared using Clustal Omega. Similarly, the remaining plasmid and *Ms*DSM14675 genes were also compared using Clustal Omega. Finally, a synteny diagram was generated using ggplot2 and geom_gene_arrow method of gggenes library in R to represent blastp alignment results among the pMF1, *Ms*CYD1, and *Ms*DSM14675.

### Comparative genomics of genus *Myxococcus* spp.

Genome sequences and associated files of 53 *Myxococcus* species and an outgroup, *Corallococcus coralloides* DSM2259, were downloaded from NCBI, and a genome statistics list with information about genome size, GC content, total contigs, total genes, and total RNAs number was tabulated (Table S2). To understand the evolutionary relationship of *Mf*124B02, *Ms*CYD1, and *Ms*DSM14675 among all 53 *Myxococcus* spp., 16S rRNA sequences with the longest length per organism were extracted. Subsequently, standalone MUSCLE (version 3.8.1551) (21) was used to run the multiple sequence alignment. Model selection for phylogeny was performed using megacc (version 7.0) (22), and then RAxML (version 8.2.12) (23) was used to build the phylogeny using the maximum likelihood method with identified GTRGAMMA model and 100 bootstrap values. RAxML bipartitions file was processed to generate the newick file, which was further visualized in the Interactive Tree of Life (iTOL) tool (24). A list of 31 housekeeping genes was used to mine homologous housekeeping genes in 53 *Myxococcus* species and 1 *Corallococcus* species, using standalone blastp. Thirty-one housekeeping genes were identified and extracted in all 54 organisms. Each gene set was subjected to multiple sequence alignment using standalone MUSCLE (version 3.8.1551) followed by sorting by headers and concatenated to create one final alignment file. The final alignment was subsequently subjected to model selection using megacc (version 7.0) followed by building the phylogeny using RAxML (maximum likelihood method, PROTGAMMALG model, 100 bootstraps). Genome to genome distance calculator (GGDC) was used to calculate the distance between all 54 genomes, thereby facilitating genome-to-genome comparison (25). The *Mf*124B02 genomic file was used as the query sequence and the reference sequence (against which the genome comparison is performed) was a set of all 53 *Myxococcus* organisms. To unravel the distance of *Ms*CYD1 with other *Myxococcus* species, GGDC was also run with *Ms*CYD1 as a query.

## RESULTS AND DISCUSSION

### Genome reannotation of pMF1

*Mf*124B02 has a complete genome assembly of 10.784 Mb with 90.46% coding density, whereas the plasmid pMF1 genome is 18.634 kb long with 85.6% coding density. NCBI annotation suggests 23 genes, of which 2 genes are present on the negative strand and the remaining 21 on the positive strand. As a single annotation might outlook a few genes, the pMF1 genome was reannotated using RAST (17) and Prokka (18), and both predicted 25 ORFs. Homology, reading frame, and relative location of all predicted ORFs were used to merge all annotations, which revealed the putative number of final annotated genes in all possible combinations (Table S1). Based on a comparative merging of all three annotations (NCBI, RAST, and Prokka), 25 putative genes or open reading frames were annotated (Table 1), which included two new open reading frames, i.e., located at 1-744 (new1) and 5540-6103 bp (new2) on the positive strand (Table S1). This reannotation-based collated information increased the plasmid coding density from 85.6% to 92.6% (Table 1).

### Investigating the evolution of plasmid sequence based on homology analysis

Blastn analysis against the PLSDB (19) indicated that the plasmid pMF1 genome has no homology with other known existing plasmids except itself. To confirm that this plasmid did not come from its host genome via duplication, plasmid protein sequences were subjected to blastp homology analysis against *Mf*124B02 encoded proteins. The results portrayed that only 4 out of 25 proteins show homology; therefore, ruling out the possibility that some portion of the complete genome might have duplicated and separated as an independent plasmid. To find any relevant homology with any available sequence, blast analysis (20) of plasmid sequences against the NR database revealed no significant hits for new1, new2, and ABX46789.1_6 whereas eight proteins namely ABX46792.1_3, ABX46792.1_9, ABX46797.1_14, ABX46798.1_15, ABX46800.1_17, ABX46801.1_18, ABX46804.1_21, and ABX46806.1_23 showed homology with the plasmid sequences itself. In this analysis, the remaining 14 genes showed homologs in either *M. stipitatus* CYD1 (*Ms*CYD1) or *M. stipitatus* DSM 14675 (*Ms*DSM14675) genomes. This analysis also revealed a few homologs within closely related organisms such as other *Myxococcus* spp., *Corallococcus*, *Cystobacter*, *Stigmatella*, and even other non-Myxococcales organisms; however, those homologs were not syntenic and were randomly present in the above-mentioned genomes.

### Homology analysis of plasmid genes suggests their putative evolution from *M. stipitatus* CYD1 via circularization of a DNA fragment

**Fig 1:**
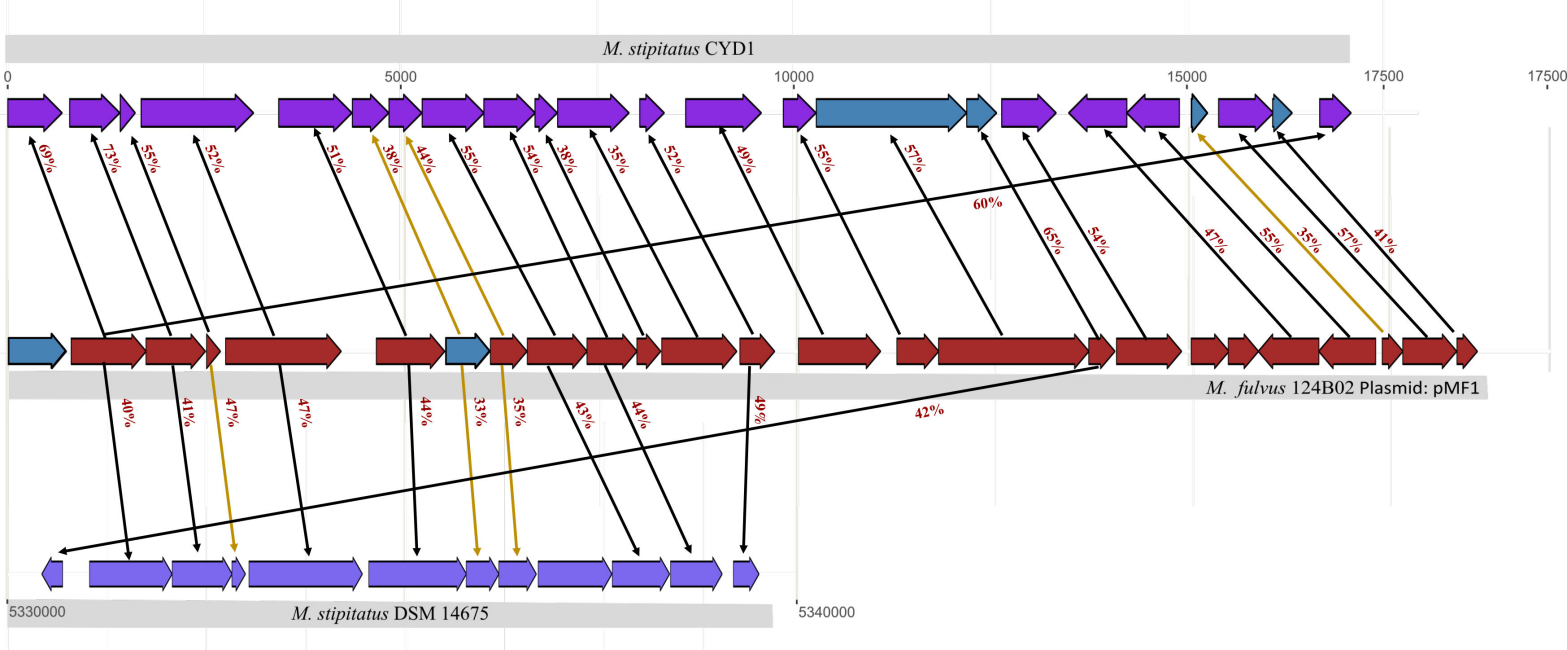
Comparative homology among the genes of plasmid *M. fulvus* 124B02 pMF1 (brick red), *M. stipitatus* CYD1 (dark purple), and *M. stipitatus* DSM14675 (light purple) depicting syntenic relationship along with percentage identity. Blue color boxes are the newly annotated genes in the pMF1 and *Ms*CYD1 genomes. Black and yellow color lines represent the percentage amino acid identity obtained using BLASTp and Clustal Omega, respectively. The respective gene IDs for all genes have been mentioned in Table S3 in the exact same order.

To confirm the homology and putative emergence from *M. stipitatus* strains, a standalone blast was specifically performed against both *M. stipitatus* strains, which revealed that out of 25 plasmid pMF1 genes, 22 and 11 genes show high identity with *Ms*CYD1 (draft genome) and *Ms*DSM14675 (complete genome) genes, respectively. These 22 and 11 genes cover the whole plasmid and approximately half of the plasmid, respectively, as depicted in Fig. 1. All 22 identified *Ms*CYD1 genes are present on a single contig (contig 28; NZ_JAKCFI010000028.1) in a syntenic manner. This contig was also further reannotated to identify any additional genes in between the already known genes. According to NCBI annotation, 19 genes were present in contig 28, whereas a hybrid annotation using RAST, Prokka, and NCBI annotation resulted in 23 genes. Intriguingly ABX46784.1_1 gene (second gene of plasmid) shows similarity with prot_3038 (first gene of the contig) as well as with prot_WP_234072500.1_3056 (last gene of the contig) (Table S3). Overall, it can be stipulated that the DNA segment corresponding to the *Ms*CYD1 contig 28 might have scissored from their ancestral genetic material, followed by circularization to form a plasmid which might have transferred in *Mf*124B02 at one point of time during evolution.

All 11 homologs in *Ms*DSM14675 are also present linearly in order, starting from the prot_WP_015349713.1_4096 gene to prot_WP_015349724.1_4107. Here, the first plasmid gene (ABX46784.1_1) shows similarity with prot_WP_015349714.1_4097 (second gene of that specific region in DSM14675), and the 17th plasmid gene (ABX46798.1_15) shows similarity with prot_WP_015349713.1_4096 gene (first gene of that particular region of DSM14675) indicating partial similarity with *Ms*DSM14675 genome (Table S3). Standalone blast analysis of *Ms*CYD1 genes against *Ms*DSM14675 revealed that all these 11 DSM14675 genes also show similarity with *Ms*CYD1 genes, which further showed similarity with the respective plasmid genes. We also found that although both strains belong to the same species, the synteny between contig 28 of *Ms*CYD1 and the respective area in *Ms*DSM14675 is highly broken. *Ms*CYD1 is a draft genome and has almost all homologs of the plasmid pMF1; however, *Ms*DSM14675, even having a complete genome, has only a few homologs. It can be argued that although there are some similarities between *Ms*CYD1 and *Ms*DSM14675 in terms of the DNA segment homologous to the pMF1 genome, *Ms*CYD1 genes and its DNA sequence corresponding to contig 28 might have contributed to the emergence of this plasmid in *Mf*124B02.

We also wanted to investigate if any type of integrases or other transfer features are present in either of the analyzed regions, both upstream and downstream. Contig 28 from *Ms*CYD1 (NZ_JAKCFI010000028) and plasmid pMF1 genome have approximately the same length (17,089 bp and 18,634 bp, respectively), and all their proteins show homology with each other (Fig. 1). Therefore, there is no way to check the nearby genes’ function in this draft assembly. We also looked for integrases or other transfer features within the complete genome of *Ms*DSM14675; however, we did not find any integrases or other transfer features upstream and downstream of the plasmid homologous sequences, i.e., WP_015349713.1_4096–WP_015349724.1_4107.

At this point, it is important to understand how similar *Ms*CYD1 and *Ms*DSM14675 are to each other at the genome and taxonomy levels, and whether the *Ms*CYD1 strain is similar to *Mf*124B02. To answer these questions, we performed comparative genomic studies among the available genus *Myxococcus* organisms.

### Phylogenetic and GGDC analyses reveal *M. stipitatus* CYD1 and *M. fulvus* 124B02 to be distinct species

**Fig 2:**
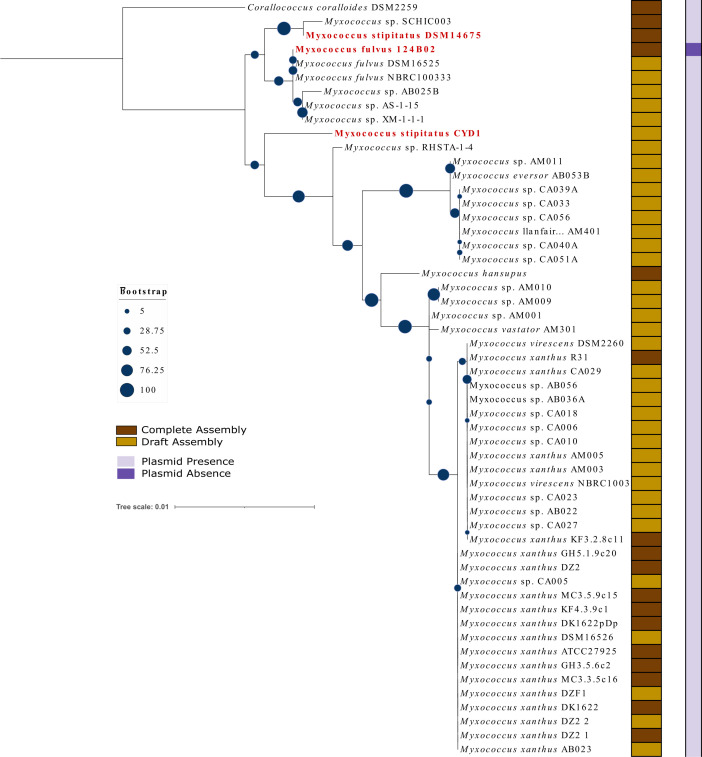
Phylogenetic tree constructed using genus *Myxococcus* spp. 16S rRNA sequences and visualized using iTOL. *Corallococcus coralloides* DSM2259 is used as an outgroup. The right-side panels depict the assembly level (complete/draft) and the presence (yes/no) of the plasmid in that organism, which is also shown in the left-side legends.

**Fig 3:**
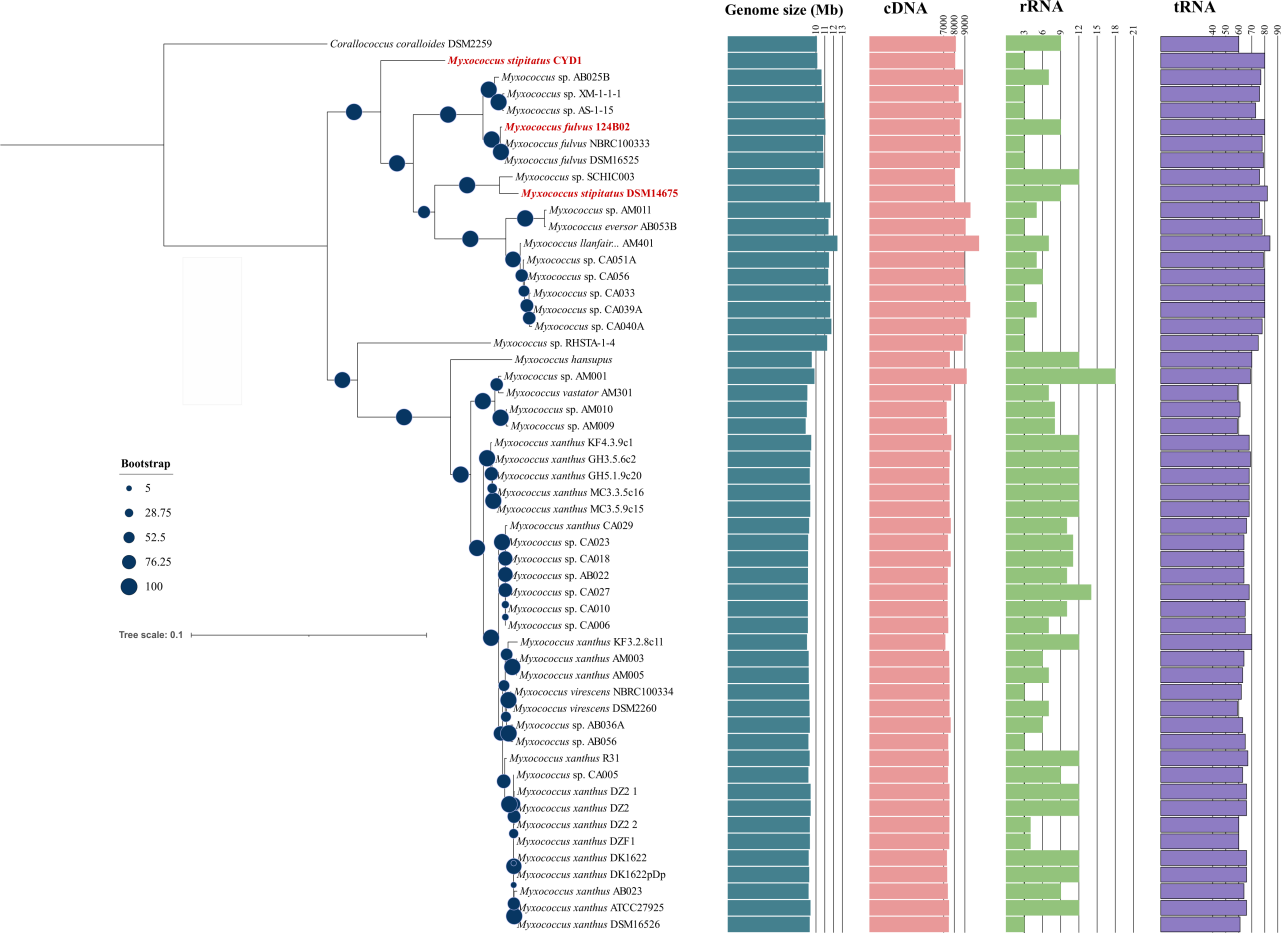
Thirty-one housekeeping gene-based phylogenetic tree: this tree has been visualized in iTOL. Right-side panels: cyan color bar plot depicts genome size, pink color bar plot shows cDNA counts, green color plot represents rRNA counts, and purple color plot represents tRNA counts per organism.

Fifty-four available genomes (53 *Myxococcus* and 1 outgroup, i.e., *Corallococcus coralloides* DSM2259) were selected for this study. *Myxococcus* spp. have genome size ranging from 8.8 Mb (*Myxococcus* sp*.* AM009) to 12.41 Mb (*M. llanfairpwllgwyngyllgogerychwyrndrobwllllantysiliogogogochensis* AM401), GC content ranging from 68% to 70% and total RNA content (including rRNA, tRNA, ncRNA, and other RNA) ranging from 68 to 95. 16S rRNA sequences of all organisms whose size ranges from 1,100 to 1,600 bp were extracted. *Corallococcus coralloides* DSM2259 was chosen as an outgroup as it belongs to the same family but is a closely related different genus. The reliability of this 16S rRNA phylogenetic tree can be confirmed as most of the *Myxococcus xanthus* and *M. fulvus* are present in their respective clades (Fig. 2). It is evident from the analysis that *Mf*124B02 and *Ms*CYD1 are not closely related as both are present in different clades. It further supports that *Ms*CYD1 and *Mf*124B02 are two different organisms. Although 16S rRNA-based phylogeny is popular and helpful, its credibility in associating taxonomic relationships between organisms below the genus level is always dubious (26). We also observed one such doubtful issue from this analysis: irrespective of belonging to the same species, *Ms*CYD1 and *Ms*DSM14675 are not present in the same clade. To understand it precisely, we opted for housekeeping genes-based phylogenetic analysis followed by genome-genome distance calculations. Similar to our previous analysis (27), 31 conserved housekeeping proteins (dnaG, frr, infC, nusA, pgK, pyrG, rplA, rplB, rplC, rplD, rplE, rplF, rplK, rplL, rplM, rplN, rplP, rplS, rplT, rpmA, rpoB, rpsB, rpsC, rpsE, rpsI, rpsJ, rpsK, rpsM, rpsS, smpB, and tsf) were used to build a concatenated protein-sequence phylogeny. This analysis revealed that all *M. fulvus* strains are closely related to each other as they all belong to the same clade (Fig. 3). *M. xanthus* and *Myxococcus virescens* are known to be similar to each other; therefore, most of the strains belonging to these two species are present in their respective clade. This study further supported that *Mf*124B02 and *Ms*CYD1 are not closely related as they belong to different clades. In concordance with 16S phylogeny, *Ms*CYD1 and *Ms*DSM14675 belong to different clades suggesting that *Ms*CYD1 might be a distinct organism compared to *M. stipitatus* and *M. fulvus*. To prove this further, we used genome-to-genome distance calculator which works based on the assumption that a DDH (DNA-DNA hybridization) value of >70 usually suggests the query organism to be of the same species. DDH calculations for *Mf*124B02 against all 53 *Myxococcus* spp. revealed that all *M. fulvus* spp. have >70 DDH values suggesting their belonging to the same species, whereas the DDH value was 26.5 and 25.3 against *Ms*DSM14675 and *Ms*CYD1, respectively, indicating that *Mf*124B02 is distantly related with *M. stipitatus*. For the *Ms*CYD1 query genome, the DDH value was 24.6 (distance: 0.1773) against *MsDSM14675*, validating that *Ms*CYD1 might be a novel species compared to *M. stipitatus* (Table S4). *Ms*CYD1 strain, with an available draft genome, has not been biochemically characterized properly until now; therefore, we request culture collection researchers and other experimental biologists to confirm further if *Ms*DSM14675 and *Ms*CYD1 share similar biochemical characteristics. If their biochemical characteristics are not similar, *Ms*CYD1 must be classified as a novel species compared to *Ms*DSM14675.

Overall, based on the relative homology of all putative ORFs between plasmid pMF1 and contig 28 of *Ms*CYD1, their syntenic arrangement of similar sequences, and taxonomic demarcation between *Ms*CYD1 and *Ms*DSM14675 based on genus *Myxococcus* phylogeny and DDH analyses, we can speculate two hypotheses in terms of pMF1 emergence:

1. Contig 28 in the *Ms*CYD1 draft genome is a part of the chromosome. Its homology within the *Ms*DSM14675 complete chromosome further supports the fact that contig 28 in the *Ms*CYD1 genome is a part of the chromosome. Based on this, it can be speculated that in the common ancestors of *Ms*CYD1 and *Mf*124B02, the segment related to contig 28 became separated from its chromosomal genetic material, got circularized, and later transferred to *Mf*124B02 as a plasmid in its course of time.
2. If complete genome sequencing of *Ms*CYD1 reveals that contig 28 is not a part of the chromosome but is a separate plasmid DNA segment, it will suggest that *Ms*CYD1 organism also harbors a plasmid, which might have been transferred to *Mf124*B02 as a plasmid itself via their common ancestors.

At present, owing to the presence of homologous syntenic segments in *Ms*CYD1, *Ms*DSM14675, and pMF1, the possibility of the first hypothesis is higher. Complete genome sequencing and experimental characterization using genetics and biochemical experiments will reveal more in-depth insights.

We would like to mention that recently another plasmid-bearing genome, *Myxococcus* sp. MxC21-1 has been sequenced and assembled (28); however, we did not find a similarity between any gene within this organism and plasmid pMF1 genes as shown in Table S5.

### Conclusion

Overall, this study first reannotated the pMF1 plasmid genome finding two additional open reading frames, further reporting that all pMF1 plasmid genes show syntenic homology within a contig of *M. stipitatus* CYD1 genome (contig 28). This evidence suggests that the *M. fulvus* 124B02 plasmid might have evolved from an *M. stipitatus* CYD1 genomic fragment. This segment is an apparent part of the chromosome as around 11 out of 25 genes are also present in the complete genome of *Ms*DSM14675, which is further supported by the fact that no plasmid-specific genes are present on it. Using 16S and housekeeping genes phylogeny and GGDC analysis, we compared all *Myxococcus* spp. to understand the relationship among *Ms*DSM14675, *Ms*CYD1, and *Mf*124B02. We further confirmed that *M. stipitatus* CYD1 is a distinct and novel species within the genus *Myxococcus*, and it should not be considered a member of *M. stipitatus*. Overall, this study discusses the solitary occurrence of a plasmid in myxobacteria showcasing its emergence from a chromosomal segment in a closely related but distinct species.

## Data Availability

Authors have used open-source tools in this analysis. All tool versions have been provided in Materials and Methods.

## SUPPLEMENTAL MATERIAL

Supplemental tables spectrum.01206-24-s0001.xlsx Tables S1 to S5.

ASM does not own the copyrights to Supplemental Material that may be linked to, or accessed through, an article. The authors have granted ASM a non-exclusive, world-wide license to publish the Supplemental Material files. Please contact the corresponding author directly for reuse.
